# Factors affecting repeated cessations of injecting drug use and relapses during the entire injecting career among the Edinburgh Addiction Cohort

**DOI:** 10.1016/j.drugalcdep.2015.03.005

**Published:** 2015-06-01

**Authors:** Yang Xia, Shaun Seaman, Matthew Hickman, John Macleod, Roy Robertson, Lorraine Copeland, Jim McKenzie, Daniela De Angelis

**Affiliations:** a MRC Biostatistics Unit, Cambridge Institute of Public Health, Forvie Site, Robinson Way, Cambridge Biomedical Campus, Cambridge CB2 0SR, UK; b School of Social and Community Medicine, University of Bristol, Canynge Hall, 39 Whatley Road, Bristol BS8 2PS, UK; c Centre for Population Health Sciences, The University of Edinburgh, Medical Quad, Teviot Place, Edinburgh EH8 9AG, UK; d Muirhouse Medical Group, 1 Muirhouse Avenue, Muirhouse, Edinburgh EH4 4PL, UK; e Public Health England, Statistics, Modelling and Economics Department, Centre for Infectious Disease Surveillance and Control, 61 Colindale Avenue, London NW9 5EQ, UK

**Keywords:** Heroin addiction, Opiate substitution treatment, Random effects model, Recurrent events, Cessation, Relapse

## Abstract

**Background and aims:**

Injecting drug use is a chronic condition, with people who inject drugs (PWID) typically experiencing repeated cessations and relapses during their injection careers. We characterize patterns of ceasing and relapsing and the impact of opiate substitution treatment (OST) during the entire injecting careers of PWID in the Edinburgh Addiction Cohort (EAC).

**Methods:**

During 2005–2007, 432 surviving participants of the EAC were interviewed about their injecting histories. Adjusted associations between covariates and hazards of cessation and relapse were estimated using random-effects models.

**Results:**

OST was strongly associated with a higher hazard of cessation (HR = 1.71, *P* < 0.001), but there was no significant evidence of association with hazard of relapse (HR = 0.81, *P* = 0.14). Women and older PWID were less likely to relapse (HR = 0.73, *P* = 0.02 and HR = 0.55, *P* < 0.001, respectively). Hazards of both cessation and relapse decreased monotonically with time since last relapse/cessation (both *P* < 0.001). An individual's hazard of cessation increased with his/her number of previous cessations (HR = 3.58 for 10+ previous cessations, *P* < 0.001), but there was no evidence that an individual's hazard of relapse changed with number of previous relapses (*P* = 0.37). There was heterogeneity in the individual hazards of both cessation and relapse.

**Conclusions:**

OST was associated with reduced time to cessation, and there was some suggestion of increased time to relapse too. The likelihood of prolonged cessation is greater for women, increases with age, and decreases with time since last relapse.

## Highlights

• We examine the pattern of ceasing and relapsing during the entire injecting career.
• Opiate substitution treatment (OST) was associated with reduced time to cessation.
• OST was associated with lower risk of relapse, but not statistically significant.
• Women and older people who inject drugs (PWID) were less likely to relapse (had lower hazard of relapse).
• With each relapse, time to next cessation event is shortened.

## 1 Introduction

Injecting drug use is an important public health problem in many countries (Mathers et al., 2008, 2010). People who inject drugs (PWID) have over 10 times greater risk of premature mortality than the general population and may contribute over 10% of deaths among young people (Bargagli et al., 2006; Degenhardt et al., 2010a). PWID are at increased risk of HIV and Hepatitis C virus infection in many countries (Alter and Moyer, 1998; De Angelis et al., 2009; Degenhardt et al., 2010b), and contribute substantially to the costs of crime and imprisonment (Godfrey and Eaton, 2002; Godfrey et al., 2004). Opiate substitution treatment is critical to reduction of drug related harm (Amato et al., 2005; van den Berg et al., 2007; Cornish et al., 2010; Degenhardt et al., 2010a; Gossop et al., 2005; Gowing et al., 2011; Turner et al., 2011) but its long-term effect on injecting cessation is uncertain.

PWID typically experience repeated periods of injecting and cessation, and have been characterized as a chronic health problem (McLellan et al., 2000; O’Brien, 2011; O’Brien and McLellan, 1996). An alternative perspective on the natural history of drug addiction has emphasized it as a problem starting in adolescence that people can ‘mature out of’ in adulthood Harding et al., 1980; Maddux and Desmond, 1980, 1986; Winick, 1962, 1964, highlighting that some individuals will cease before becoming dependent or only after a short period of injecting (Biernacki, 1986; Robbins et al., 1975; Sweeting et al., 2009; Zinberg and Jacobson, 1976).

The duration of injecting, likelihood of long-term cessation and the factors that promote cessation and recovery are important both to policy on drug treatment and assessments of disease burden, but these quantities remain uncertain. In part this is because long-term follow-up and natural history studies of opiate and injecting drug use are rare. In this study we examine patterns of ceasing and relapsing during the entire injecting career, and explore associations between opiate substitution treatment and other covariates and risks of recovery and relapse.

## 2 Material and methods

### 2.1 Data source

The Edinburgh Addiction Cohort (EAC) is a prospective open cohort study of 794 opiate injectors recruited by Muirhouse Medical Group from 1980 to 2006 (Kimber et al., 2010; Macleod et al., 2010; Robertson et al., 1994; Skidmore et al., 1990). Information on opiate substitution treatment (OST) use (methadone, buprenorphine or dihyrocodeine) and age at onset was extracted from primary care case notes (Cornish et al., 2010; Robertson et al., 2006). In 2005–2007, the surviving participants were interviewed about their past patterns of injecting. A full account of this cohort is given elsewhere (Copeland et al., 2004; Macleod et al., 2010; Robertson et al., 1986; Robertson and Richardson, 2007). Case notes were available for 655 (83%) individuals (22 had no contact details, 30 not traced, 38 no response, 40 declined, 9 too ill). Of these, 223 had died before interview and 27 others had missing year of onset of injection. Here we focus on the 405 remaining individuals. For these 405 individuals, the mean (standard deviation, SD) number of years from recruitment to interview was 10.8 (7.2) and that from year of first injection to interview was 17.6 (9.0).

During the interviews, individuals filled out retrospective life grids indicating, for each calendar year since beginning to inject, whether they had injected in that year. For each year that an individual reported injecting, the individual was asked whether there was a period lasting at least 3 months in that year during which he/she did not inject (a ‘non-injection period’). If there was such a period, the individual was asked to estimate the number of distinct such non-injection periods and the total number of days spent injecting in that year (Macleod et al., 2010). Periods of abstinence that lasted less than 3 months were not elicited. Interviews were anchored around memorable events (e.g. death of father, divorce) to aid recall (Macleod et al., 2010).

### 2.2 Cessation and relapse times

**Fig. 1:**
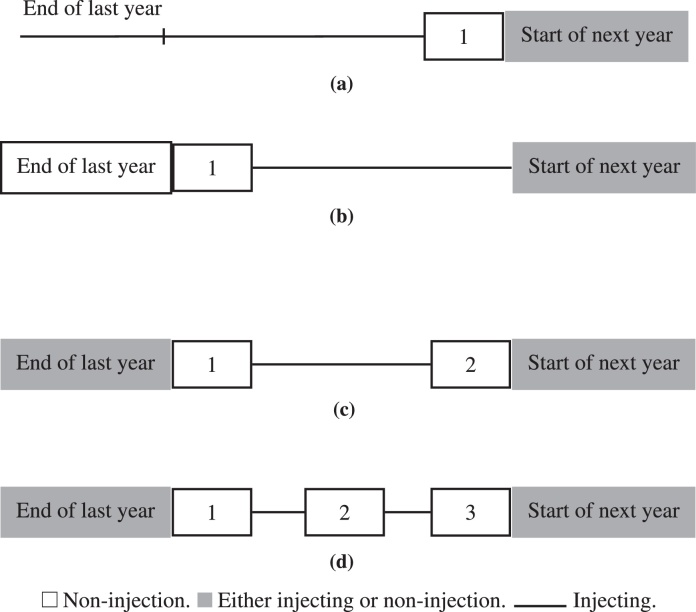
The assignment of non-injection periods for the 405 PWID in the EAC, United Kingdom, 1980–2006. (a) A single period of non-injection preceded by a period of injecting. (b) A single period of non-injection preceded by a period of non-injection. (c) Two non-injection periods within 1 year. (d) Three non-injection periods within 1 year.

An injection career can be thought of as a sequence of recurrent events in which the injector switches repeatedly between injection and non-injection. We define a ‘cessation’ as the beginning of a non-injection period, and define a ‘relapse’ as the end of such a period. The data available from the interview questionnaires did not determine precisely when cessations and relapses occurred. So instead we used the following algorithm to impute these times. This algorithm aims to minimize the number of cessations and relapses, by assuming, whenever possible, that two periods of non-injecting reported in consecutive calendar years corresponded to a single continuous period of non-injecting spanning the new year. Fig. 1 illustrates the algorithm.

For each individual, the algorithm begins by taking each calendar year in turn, in chronological order, and assigning non-injection periods in that year to the beginning, middle or end of the year. When there is only one non-injection period in the year, it is placed at the end of that year if the preceding year ended with an injecting period (Fig. 1a), and at the beginning of the year otherwise (Fig. 1b). When there are two non-injection periods, one is put at the beginning of the year and one at the end (Fig. 1c). When there are three non-injection periods, they are put at the beginning, middle, and end (Fig. 1d). The exact beginning and end times of these non-injecting periods (i.e. times of cessation and relapse) are then imputed by partitioning the total number of days that the individual reported not injecting in that calendar year equally between the 1–3 non-injecting periods.

### 2.3 Statistical analysis

The Kaplan–Meier estimator was used to estimate the distribution of time from injection onset to first cessation and of the subsequent time to the next relapse.

Random-effects proportional-hazards models were used to investigate the dependence of the hazards of cessation and relapse on covariates, accounting for correlation between repeated times to event (cessation or relapse) within the same individual (Aalen et al., 2008). The time-constant covariates were sex, age at injection onset, and year of injection onset (before or after 1986); the time-varying covariates were current OST use, number of previous cessations/relapses, and current age. OST was a time-varying three-level categorical variable taking values ‘currently prescribed OST’, ‘not currently prescribed OST’ or ‘unknown whether currently prescribed OST’ (or ‘on OST’, ‘off OST’ and ‘unknown OST’ for short). Year of injection onset was dichotomized at 1986 because this is when HIV test became widely available, which may have altered injecting behavior. Data on all these covariates except OST use were obtained during the interview.

The model for relapse is as follows (that for cessation is analogous). Let *h*_*ij*_(*t*) denote the hazard of relapse for individual *i* at time *t* during his/her *j*th non-injection period (where time zero means beginning of that period). Let OST_on,*ij*_(*t*) equal 1 if individual *i* is on OST at that time, and 0 otherwise. Similarly, let OST_na,*ij*_(*t*) equal 1 if the individual has unknown OST status at that time, and 0 otherwise, and let ***Z***_*ij*_(*t*) denote the values of the remaining covariates (sex, current age, etc.) at that time. Then a basic random-effects proportional hazards model is given by

$$h_{\mathrm{ij}}(t)=h_{0}(t)\exp[\beta_{\mathrm{on}}{\mathrm{OST}}_{\mathrm{on},\mathrm{ij}}(t)+\beta_{\mathrm{na}}{\mathrm{OST}}_{\mathrm{na},\mathrm{ij}}(t)+\beta_{Z}Z_{\mathrm{ij}}(t)+u_{i}]$$

where *h*_0_(*t*) is the baseline hazard, and *u*_*i*_ is a random-effect specific to individual *i*. The random effects are assumed to be normally distributed with mean zero. A positive random effect *u*_*i*_ implies that individual *i* has a greater baseline hazard of relapse than the average individual; negative *u*_*i*_ implies a lower than average hazard of relapse. Parameters *β*_on_ and *β*_na_ are the log hazard ratios of relapse when on OST and unknown OST, respectively, compared to when off OST; and ***β***_*Z*_ are the log hazard ratios of the remaining covariates. Log hazard ratios greater than zero (or, equivalently, hazard ratios greater than one) imply a shorter expected time to relapse, and so shorter non-injection period; log hazard ratios less than zero (equivalently, hazard ratios less than one) imply a longer expected time to relapse, and so longer non-injection period.

The baseline hazard *h*_0_(*t*) for relapse was assumed to be piecewise-constant over four intervals: ≤2 months, (2, 12] months, (1, 2] years, and more than 2 years. For cessation, five intervals were used: ≤3 days, (3, 7] days, (1, 12] weeks, (3, 12] months, and more than 1 year. These change points were chosen by examining plots of the cumulative hazards. This procedure was further aided by comparing nested models with increasing number of change points using likelihood ratio tests so that non-significant change points could be dropped.

A potential problem with this basic model is ‘confounding by cluster’ (Seaman et al., 2014). The basic model above assumes that the probability that individual *i* is on OST at any given time *t* (*OST*_on,*ij*_(*t*) = 1) is uncorrelated with his/her random effect *u*_*i*_. In practice, however, it is possible that an individual with a high random effect for relapse (and so who tends to relapse quickly after cessation) may be more likely to be prescribed OST than another individual who has a lower random effect (and so who tends to relapse slower). This can cause difficulties with both interpretation and estimation of the OST effect parameters *β*_on_ and *β*_na_ (Seaman et al., 2014). To deal with this potential problem of confounding by cluster, we used the Poor Man's method of Neuhaus and Kalbfleisch (1998). This involves separating each of the OST effects *β*_on_ and *β*_na_ into a between-individual and a within-individual effect, which is achieved by replacing the basic model above with

$$\begin{array}{ccc} h_{\mathrm{ij}}(t) & = & h_{0}(t)\exp[\beta_{\mathrm{on}}^{B}{\bar{\mathrm{OST}}}_{\mathrm{on},i}+\beta_{\mathrm{on}}^{W}\{{\mathrm{OST}}_{\mathrm{on},\mathrm{ij}}(t)-{\bar{\mathrm{OST}}}_{\mathrm{on},i}\}\\ & & +\beta_{\mathrm{na}}^{B}{\bar{\mathrm{OST}}}_{\mathrm{na},i}+\beta_{\mathrm{na}}^{W}\{{\mathrm{OST}}_{\mathrm{na},\mathrm{ij}}(t)-{\bar{\mathrm{OST}}}_{\mathrm{na},i}\}+\beta_{Z}Z_{\mathrm{ij}}(t)+u_{i}] \end{array}$$

where ${\bar{\mathrm{OST}}}_{\mathrm{on},i}$ and ${\bar{\mathrm{OST}}}_{\mathrm{na},i}$ denote the fractions of individual *i*'s total follow-up spent on OST and with unknown OST, respectively. The parameter $\beta_{\mathrm{on}}^{B}$ describes the between-individual effect of being on OST, i.e. the log hazard ratio comparing *two different individuals*: the average individual who spends all his/her time on OST and the average individual who spends no time on OST. The parameter $\beta_{\mathrm{on}}^{W}$ describes the within-individual effect of being on OST, i.e. the log hazard ratio of *the same individual* at two different times: when on OST and when off OST. The between- and within-individual effects of having unknown OST, $\beta_{\mathrm{na}}^{B}$ and $\beta_{\mathrm{na}}^{W}$, are analogous. Within-individual effects are of more interest than between-individual effects, because individuals who spend a lot of time on OST may differ in many unmeasured ways from individuals who spend little time on OST.

Finally, we carried out two sensitivity analyses. First, we fitted a Cox model with random effects, in order to avoid assuming a piecewise-constant form for *h*_0_(*t*). Second, we fitted a joint model for cessation and relapse, allowing the random effects for cessation and relapse to be correlated. This enabled us to check that such a correlation was not causing bias due to violation of our model's independent censoring assumption (Xia, 2013). The hazard ratio estimates and *P* values for OST and other covariates obtained from both these models were similar to those from the model described above, and so we do not report them here. The models with piecewise-constant hazard were fitted by maximum likelihood in STATA (StataCorp, 2009) and, for the joint model, in R using bespoke code (Core Team, 2014); the Cox model with random effects was fitted using the coxme package in R (Therneau, 2012).

## 3 Results

**Fig. 2:**
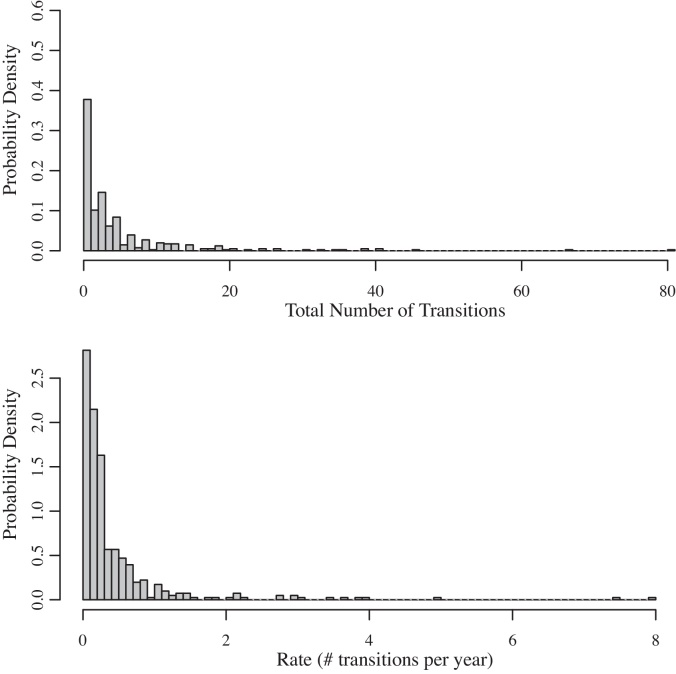
Histograms of the total number and rate of transitions for the 405 PWID in the EAC, United Kingdom, 1980–2006.

Fig. 2 (top) shows the distribution of the total number of transitions (either cessation or relapse) per individual during the follow-up period. The median (interquartile range, IQR) of the total number of transitions (either cessation or relapse) per individual during the follow-up period was 3 (1–4); the mean (SD) was 5 (9) and the maximum was 81. Eighteen (4%) individuals made no transitions, i.e. they injected throughout follow-up; 65 (16%) injected for less than 1 year with no further relapse; and 252 (65%) experienced at least one relapse. Fig. 2 (bottom) shows the distribution of the total number of transitions per individual divided by his/her years of follow-up.

### 3.1 Kaplan–Meier curves

**Fig. 3:**
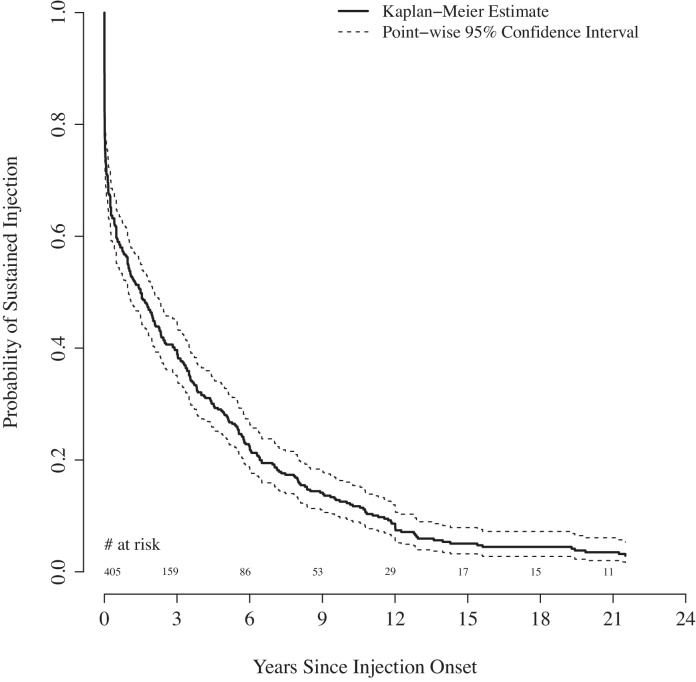
Kaplan–Meier estimate of time to first cessation for the 405 PWID in the EAC, United Kingdom, 1980–2006.

**Fig. 4:**
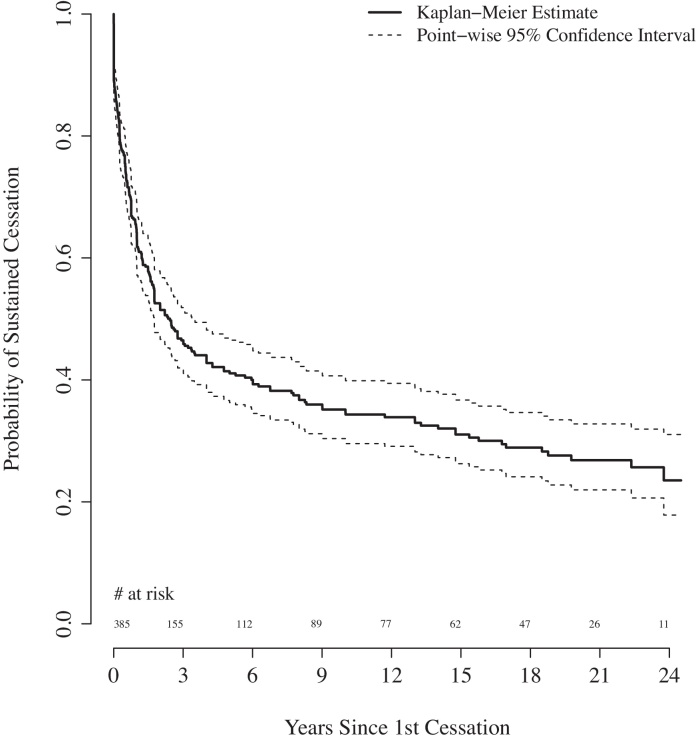
Kaplan–Meier estimate of time to first relapse for the 405 PWID in the EAC, United Kingdom, 1980–2006.

The Kaplan–Meier estimated probability that a first-time injector ceased within 12 months was 0.46 (95% CI: 0.41, 0.50) (Fig. 3), and within 5 years was 0.72 (95% CI: 0.67, 0.76). The estimated median (IQR) time to first cessation was 1.5 (0.0–5.6) years. The estimated probability of subsequent relapse was 0.37 (95% CI: 0.32, 0.42) at 12 months and 0.59 (95% CI: 0.53, 0.63) at 5 years (Fig. 4). The estimated median (IQR) time to subsequent relapse was 2.4 (0.5–23.8) years.

### 3.2 Regression models

Table 1 shows the estimated hazard ratios from the proportional hazards models with piecewise-constant baseline hazards. Opiate substitution treatment (OST) use was associated with higher hazard of cessation within an individual (HR = 1.71 for on OST, 95% CI: 1.40, 2.09, overall *P* < 0.001 from likelihood-ratio test on 2 degrees of freedom). The between-individual effect of OST provides evidence of confounding (with hazard ratios in the opposite direction to within-individual effects), suggesting that those people who cease slower may also be those more likely to be prescribed OST. An individual's hazard of cessation increased with his/her number of previous cessations (overall *P* < 0.001): compared to the first injection period, the hazard was 1.19 times as large (95% CI: 0.97, 1.47) during the second and third periods, and was 3.58 times as large (95% CI: 2.42, 5.29) after the 10th period. People who inject drugs (PWID) who began injecting after 1986 had a higher hazard (HR = 1.37, 95% CI: 1.05, 1.80, *P* = 0.02). There was weak evidence that PWID who began injecting at an older age (≥20) had a higher hazard of cessation (HR = 1.32, 95%CI: 0.99, 1.76, *P* = 0.06). No evidence of the hazard differing by gender or current age has been found.

For hazard of relapse, there was some suggestion of a within-individual effect of OST (HR = 0.81 for on OST), but this was non-significant (overall *P* = 0.14). The between-individual effect of OST (HR = 1.67 for on OST, overall *P* = 0.007) was significant, which is probably again due to confounding: those PWID who relapse faster are also those who are more likely to be prescribed OST. Women had a lower hazard of relapse (HR = 0.73, 95% CI: 0.56, 0.94, *P* = 0.02). Current age was also significantly associated with hazard (overall *P* = 0.001), with the hazard after age 35 years dropping to 55% of that at age <20 years (HR = 0.55, 95% CI: 0.41, 0.75). PWID who began injecting at an older age (≥20) had a higher hazard of relapse (HR = 1.40, 95% CI: 1.08, 1.83, *P* = 0.01). There was no evidence that an individual's hazard differed by calendar period of onset or changed as the number of his/her previous relapses increased.

The hazards of both cessation and relapse were monotonically decreasing with time since last relapse/cessation (overall *P* < 0.001 for both). The hazard of cessation during (3, 7] days since last relapse was only 0.51 (95% CI: 0.40, 0.64) times the hazard during [1, 3] days. Thereafter the hazard continued to decline, with the hazard ratio reaching 0.03 after 1 year (95% CI: 0.03, 0.04) compared to that during [1, 3] days. The hazard of relapse (1, 2] years after most recent cessation was only 0.38 (95% CI: 0.30 and 0.50) times the hazard after ≤2 months; after 2 years this hazard ratio had decreased to 0.10 (95% CI: 0.08, 0.13).

We found evidence for significant heterogeneities in the hazards of cessation and relapse between individuals: variances of random effects were 0.90 (95% CI: 0.65, 1.24, *P* < 0.001) and 0.57 (95% CI: 0.33, 0.97, *P* < 0.001), respectively.

## 4 Discussion

We found evidence of a strong inverse association between being on opiate substitution treatment (OST) and the average duration of injection episodes. There was insufficient evidence, however, that OST exposure reduced the risk of relapse. Women had lower rates of relapse than men. Age was positively associated with lower rates of relapse but not with time to cessation. An individual's rate of cessation increased as he/she accumulated more previous cessations, but there was little evidence that his/her rate of relapse changed as he/she accumulated more previous relapses. The risk of cessation or relapse is not constant but decreases with time elapsed since the previous relapse or cessation, respectively. For example, the risk of relapse after more than 2 years of non-injecting was 10% of the risk at the start of a period of non-injecting. Thus, the likelihood that an individual experiences long-term cessation, though not explicitly modeled, increases with age, number of previous cessation events, and the duration of non-injecting. There was evidence of heterogeneity in the individual risks of both cessation and relapse.

### 4.1 Strengths and limitations

The Edinburgh Addiction Cohort (EAC) features a long period of follow-up (median 18.5 years). This has enabled us to examine long-term injecting patterns and to extend the analysis of patterns of injecting beyond first cessation and relapse.

However, our findings are subject to several limitations and potential biases. First, the cohort may under-represent people who inject drugs (PWID) for very short periods, because they may be less likely to experience health or other problems and therefore less likely to come to the attention of primary care. EAC participants were enrolled on the basis that they report injecting drug use or present with drug related problems in primary care; and though time from onset to recruitment was shorter for EAC than for many other cohorts (Macleod et al., 2010) there is likely to be some selection bias. For instance, approximately 16% of EAC report injecting periods of less than 1 year, which is slightly lower than the proportion estimated from other studies of between 20% and 50%  (Sweeting et al., 2009), suggesting the potential for under-ascertainment.

Second, there is likely to be survivor bias, as information on transitions between injection and non-injection is available only for those who survived long enough to attend the interview. Unfortunately, the information from clinical notes on injecting patterns is not sufficiently complete to allow periods of injecting/non-injecting to be identified for the deceased cases (Macleod et al., 2012). The times to cessation and relapse and the impact of the covariates on these times, therefore, may differ for people who have not survived. Although the model presented in this paper does not include a long-term cessation state, we showed earlier that the effect of covariates on time to long-term cessation was not biased by excluding deceased cohort participants (Kimber et al., 2010).

Third, there also may be recall bias, since the data on these transitions were collected retrospectively through a single questionnaire. We tried addressing the recall bias by including a categorical variable for decades (e.g. 1960s, 70s, 80s, etc.) in the regression model; the regression results were little affected and hence this variable for decades was excluded in the model presented.

Fourth, cessation and relapse times were imputed from the interview data using the algorithm described in Section 2, because data on injecting periods were limited to the number of non-injecting periods in each calendar year. The algorithm minimizes the number of transitions, which may have caused the hazards of cessation and relapse to be underestimated. In addition, some of the cessations and relapses that we imputed as occurring when PWID were on OST may actually have occurred when they were off OST, and vice versa. This misclassification could cause bias in the estimated hazard ratios of OST. However, there are two reasons to believe that this will have little impact on our conclusions. Firstly, the data on OST use came from the case notes, rather than from interviews, and the method of imputing cessation/relapse times made no use of these OST data. Thus, any misclassification would be expected to dilute any true association between OST use and hazard of cessation or relapse, rather than to create an apparent association where none exists. Secondly, the main factor determining the estimated hazard ratios of OST is the total numbers of cessations/relapses occurring while on OST, off OST and unknown OST. 94% of both the imputed cessations and the imputed relapses took place in calendar years during which PWID were entirely on OST, entirely off OST or had entirely unknown OST status. For these cessations and relapses, moving the imputed time to earlier or later in the year would not change whether they occurred when on, off or unknown OST, and so would not change the total numbers of cessations/relapses occurring while on OST, off OST and unknown OST.

Fifth, biases could have been introduced to the associations presented in Table 1 due to confounding by omitted time-dependent behavioral covariates, such as incarceration history. These data were poorly recorded in primary care notes and also unavailable through record linkage (Macleod et al., 2012).

### 4.2 Implications and other evidence

Out of the few longitudinal studies of injecting heroin use, most have tended to emphasize the persistence and high rates of relapse and high mortality rates associated with injecting heroin use (Galai et al., 2003; Goldstein and Herrera, 1995; Hser et al., 2001; Kimber et al., 2010; Rathod et al., 2005; Stimson and Oppenheimer, 1982; Termorshuizen et al., 2005a; Vaillant, 1973). Only a few studies have tried to characterize the injection career and explored factors that may influence injecting duration, notably the Amsterdam Cohort Study (ACS), the AIDS Link to Intravenous Experience (ALIVE) cohort in Baltimore and California Civil Addict Program (CAP) (Galai et al., 2003; Hser et al., 2007; Nosyk et al., 2013; Shah et al., 2006; Termorshuizen et al., 2005b; Vlahov et al., 1991). The ALIVE and ACS cohorts both have reported high rates of cessation and relapse during follow-up and that OST was associated with a faster time to cessation. For example, Shah et al. (2006) found that 86% of the non-injection/occasional use periods were followed by relapse within 5 years and estimated median time to cessation and relapse was 4 and 1 year respectively. Termorshuizen et al. (2005b) in an analysis of the ACS also found that OST was associated with longer injecting careers and did not appear to promote ‘long-term cessation’. In addition, Nosyk et al. (2013) assessing the CAP showed that long-term cessation or sustained abstinence often occurs after multiple periods of recovery and relapse, and that previous number of abstinent events and age are associated with duration of abstinence and time to next recovery event.

Galai et al. (2003) described several classes of injector based on persistence and number of relapses and found that group membership was associated with history of incarceration, age, and OST exposure. However, their classification of injecting patterns could potentially bias the inferences, as length of follow-up may be a confounder for the relationship between the risk factors and injecting group membership. In addition, these previous analyses examine time to cessation after recruitment rather than from injecting onset which our analyses suggest may introduce bias since number of previous cessations and age are associated with time to cessation and hazard of relapse respectively. It has been argued that a lack of association between injecting patterns and age or episode number is consistent with the thesis that addiction is a ‘chronic relapsing problem’ rather than a problem ‘that people mature out of’ (Langendam et al., 2000; McLellan et al., 2000; Termorshuizen et al., 2005b; van den Berg et al., 2007; Winick, 1962). We do find evidence of an association between cessation and relapse with age and episode number. However, very few of our cohort members remain ‘untreated’ and therefore can be considered as ceasing ‘naturally’ (if this is construed as without treatment) and so our findings also are consistent with a chronic relapsing disease.

## Role of funding source

The Edinburgh Addiction Study has been previously supported by the Scottish Home and Health Department and the Medical Research Council. The present follow-up study is supported by the Chief Scientists Office for Scotland (grant nos. CZH/4/318 and CZH/4/40). The funders had no role in the design, execution, and writing up of the study.

## Contributors

Yang Xia

- analysis and interpretation of data
- drafting the article, revising the article critically for important intellectual content
- final approval of the version to be published

Shaun Seaman

- conception and design, analysis and interpretation of data
- revising the article critically for important intellectual content
- final approval of the version to be published

Matthew Hickman

- conception, design, and obtaining funding
- interpretation of data
- revising the article critically for important intellectual content
- final approval of the version to be published

John Macleod

- conception, design, and obtaining funding
- acquisition and interpretation of data
- reviewing the article critically for important intellectual content
- final approval of the version to be published

Roy Robertson

- conception, design, and obtaining funding
- acquisition and interpretation of data
- reviewing the article critically for important intellectual content
- final approval of the version to be published

Lorraine Copeland

- acquisition of data
- reviewing the article
- final approval of the version to be published

James McKenzie

- acquisition of data
- reviewing the article
- final approval of the version to be published

Daniela De Angelis

- conception and design, analysis and interpretation of data
- revising the article critically for important intellectual content
- final approval of the version to be published

## Conflict of interest

No conflict declared.

## Figures and Tables

**Table 1:** Estimated associations between covariates and hazards of cessation and relapse among the EAC, United Kingdom, 1980–2006.

	Cessation			Relapse		
	HR^a^	95% CI^a^	*P*-value	HR	95% CI	*P*-value
Sex						
Male (Ref)	1.00			1.00		
Female	1.05	0.81, 1.36	0.71	0.73	0.56, 0.94	0.02
Age at injection onset						
12–19 (Ref)	1.00			1.00		
20+	1.32	0.99, 1.76	0.06	1.40	1.08, 1.83	0.01
Year of injection onset						
<1986 (Ref)	1.00			1.00		
≥1986	1.37	1.05, 1.80	0.02	1.22	0.94, 1.58	0.14
Current age			0.62			0.001
<20	0.94	0.74, 1.18	0.59	1.06	0.78, 1.44	0.73
20–25 (Ref)	1.00				1.00	
26–30	0.96	0.79, 1.16	0.66	0.70	0.57, 0.87	0.001
31–35	1.10	0.87, 1.39	0.41	0.77	0.61, 0.98	0.04
35+	1.15	0.85, 1.56	0.37	0.55	0.41, 0.75	<0.001
OST exposure						
Between-individual			<0.001			0.007
Not on OST (Ref)	1.00			1.00		
On OST	0.73	0.53, 1.02	0.07	1.67	1.16, 2.40	0.006
Unknown	0.35	0.21, 0.57	<0.001	1.95	1.18, 3.23	0.01
Within-individual			<0.001			0.14
Not on OST (Ref)	1.00			1.00		
On OST	1.71	1.40, 2.09	<0.001	0.81	0.65, 1.00	0.05
Unknown	1.11	0.81, 1.54	0.52	0.83	0.59, 1.18	0.30
No. of previous cessations/relapses			<0.001			0.37
0 (Ref)	1.00			1.00		
1–2	1.19	0.97, 1.47	0.09	1.27	1.00, 1.62	0.05
3–4	1.52	1.14, 2.03	0.005	1.36	0.96, 1.92	0.09
5–9	2.67	1.94, 3.69	<0.001	1.44	0.97, 2.14	0.07
≥10	3.58	2.42, 5.29	<0.001	1.54	0.96, 2.48	0.08
Baseline hazard for cessation			<0.001			
≤3 days (Ref)	1.00					
(3, 7] days	0.51	0.40, 0.64	<0.001			
(1, 12] weeks	0.09	0.08, 0.11	<0.001			
(3, 12] months	0.04	0.03, 0.05	<0.001			
>1 year	0.03	0.03, 0.04	<0.001			
Baseline hazard for relapse						<0.001
≤2 months (Ref)				1.00		
(2, 12] months				0.87	0.74, 1.03	0.11
(1, 2] years				0.38	0.30, 0.50	<0.001
>2 years				0.10	0.08, 0.13	<0.001

Variance of log-normal random effects	0.90	0.65, 1.24	<0.001	0.57	0.33, 0.97	<0.001

a CI, confidence interval; HR, hazard ratio.

## References

[bib0005] Aalen O.O., Borgan Ø, Gjessing H.K. (2008). Survival and Event History Analysis – A Process Point of View.

[bib0010] Alter M., Moyer L. (1998). The importance of preventing hepatitis C virus infection among injection drug users in the United States. J. Acquir. Immune Defic. Syndr. Hum. Retrovirol..

[bib0015] Amato L., Davoli M., Perucci C., Ferri M., Faggiano F., Mattick R. (2005). An overview of systematic reviews of the effectiveness of opiate maintenance therapies: available evidence to inform clinical practice and research. J. Subst. Abuse Treat..

[bib0020] Bargagli A., Hickman M., Davoli M., Perucci C., Schifano P., Buster M. (2006). Drug-related mortality and its impact on adult mortality in eight European countries. Eur. J. Public Health.

[bib0025] Biernacki P. (1986). Pathways from Heroin Addiction: Recovery Without Treatment.

[bib0030] Copeland L., Budd J., Elton R. (2004). Changing patterns in causes of death in a cohort of injecting drug users, 1980–2001. Arch. Intern. Med..

[bib0035] Cornish R., Macleod J., Strang J., Vickerman P., Hickman M. (2010). Risk of death during and after opiate substitution treatment in primary care: prospective observational study in UK General Practice Research Database. BMJ.

[bib0040] De Angelis D., Sweeting M., Ades A., Hickman M., Hope V., Ramsay M. (2009). An evidence synthesis approach to estimating hepatitis C prevalence in England and Wales. Stat. Methods Med. Res..

[bib0045] Degenhardt L., Bucello C., Mathers B., Briegleb C., Ali H., Hickman M. (2010). Mortality among regular or dependent users of heroin and other opioids: a systematic review and meta-analysis of cohort studies. Addiction.

[bib0050] Degenhardt L., Mathers B., Vickerman P., Rhodes T., Latkin C., Hickman M. (2010). Prevention of HIV infection for people who inject drugs: why individual, structural, and combination approaches are needed. Lancet.

[bib0055] Galai N., Safaeian M., Vlahov D., Bolotin A., Celentano D.D. (2003). Longitudinal patterns of drug injection behavior in the ALIVE Study cohort, 1988–2000: description and determinants. Am. J. Epidemiol..

[bib0060] Godfrey C., Eaton G. (2002). The economic and social costs of Class A drug use in England and Wales, 2000. Technical Report.

[bib0065] Godfrey C., Stewart D., Gossop M. (2004). Economic analysis of costs and consequences of the treatment of drug misuse: two year outcome data from the National Treatment Outcome Research Study (NTORS). Addiction.

[bib0070] Goldstein A., Herrera J. (1995). Heroin addicts and methadone treatment in Albuquerque: a 22-year follow-up. Drug Alcohol Depend..

[bib0075] Gossop M., Trakada K., Stewart D., Witton J. (2005). Reductions in criminal convictions after addiction treatment: 5-year follow-up. Drug Alcohol Depend..

[bib0080] Gowing L., Farrell M., Bornemann R., Sullivan L., Ali R. (2011). Oral substitution treatment of injecting opioid users for prevention of HIV infection. Technical Report 8. Cochrane Database Syst. Rev..

[bib0085] Harding W., Zinberg N., Stelmack S., Barry M. (1980). Formerly-addicted-now-controlled opiate users. Int. J. Addict..

[bib0090] Hser Y., Huang D., Chou C., Anglin M. (2007). Trajectories of heroin addiction: growth mixture modeling results based on a 33-year follow-up study. Eval. Rev..

[bib0095] Hser Y.I., Hoffman V., Grella C.E., Anglin M.D. (2001). A 33-year follow-up of narcotics addicts. Arch. Gen. Psychiatry.

[bib0100] Kimber J., Copeland L., Hickman M., Macleod J., McKenzie J., De Angelis D., Robertson J.R. (2010). Survival and cessation in injecting drug users: prospective observational study of outcomes and effect of opiate substitution treatment. BMJ.

[bib0105] Langendam M.W., Van Brussel G.H.A., Coutinho R.A., Van Ameijden E.J.C. (2000). Methadone maintenance and cessation of injecting drug use: results from the Amsterdam Cohort Study. Addiction.

[bib0110] Macleod J., Copeland L., Hickman M., McKenzie J., Kimber J., De Angelis D., Robertson J.R. (2010). The Edinburgh Addiction Cohort: recruitment and follow-up of a primary care based sample of injection drug users and non drug-injecting controls. BMC Public Health.

[bib0115] Macleod J., Hickman M., Jones H.E., Copeland L., McKenzie J.S., De Angelis D. (2012). Early life influences on the risk of injecting drug use: case control study based on the Edinburgh Addiction Cohort. Addiction.

[bib0120] Maddux J., Desmond D. (1980). New light on the maturing out hypothesis in opioid dependence. Bull. Narc..

[bib0125] Maddux J., Desmond D. (1986). Relapse and recovery in substance abuse careers. NIDA Res. Monogr..

[bib0130] Mathers B., Degenhardt L., Ali H., Wiessing L., Hickman M., Mattick R. (2010). HIV prevention, treatment, and care services for people who inject drugs: a systematic review of global, regional, and national coverage. Lancet.

[bib0135] Mathers B., Degenhardt L., Wiessing L., Hickman M., Strathdee S., Wodak A. (2008). Global epidemiology of injecting drug use and HIV among people who inject drugs: a systematic review. Lancet.

[bib0140] McLellan A., Lewis D., O’Brien C., Kleber H. (2000). Drug dependence, a chronic medical illness: implications for treatment, insurance and outcomes evaluation. JAMA.

[bib0145] Neuhaus J.M., Kalbfleisch J.D. (1998). Between- and within-cluster covariate effects in the analysis of clustered data. Biometrics.

[bib0150] Nosyk B., Anglin M.D., Brecht M.L., Lima V.D., Hser Y.I. (2013). Characterizing durations of heroin abstinence in the California Civil Addict Program: results from a 33-year observational cohort study. Am. J. Epidemiol..

[bib0155] O’Brien C.P. (2011). Addiction and dependence in DSM-V. Addiction.

[bib0160] O’Brien C.P., McLellan A.T. (1996). Myths about the treatment of addiction. Lancet.

[bib0165] R Core Team (2014). R: A Language and Environment for Statistical Computing. http://www.R-project.org/.

[bib0170] Rathod N., Addenbrooke W., Rosenbach A. (2005). Heroin dependence in an English town: 33-year follow-up. Br. J. Psychiatry.

[bib0175] Robbins L., Helzer J., Davies D.H. (1975). Narcotic use in southeast-Asia and afterward – interview study of 898 Vietnam returnees. Arch. Gen. Psychiatry.

[bib0180] Robertson J., Bucknall A., Welsby P., Roberts J., Inglis J., Peutherer J., Brettle R. (1986). Epidemic of AIDS related virus (HTLV-III/LAV) infection among intravenous drug abusers. BMJ.

[bib0185] Robertson J.R., Raab G.M., Bruce M., McKenzie J.S., Storkey H.R., Salter A. (2006). Addressing the efficacy of dihydrocodeine versus methadone as an alternative maintenance treatment for opiate dependence. A randomized controlled trial. Addiction.

[bib0190] Robertson J.R., Ronald P.J., Raab G.M., Ross A.J., Parpia T. (1994). Deaths, HIV infection, abstinence, and other outcomes in a cohort of injecting drug users followed up for 10 years. BMJ.

[bib0195] Robertson R., Richardson A. (2007). Heroin injecting and the introduction of HIV/AIDS into a Scottish city. J. R. Soc. Med..

[bib0200] Seaman S., Pavlou M., Copas A. (2014). Review of methods for handling confounding by cluster and informative cluster size in clustered data. Stat. Med..

[bib0205] Shah N.G., Galai N., Celentano D.D., Vlahov D., Strathdee S.A. (2006). Longitudinal predictors of injection cessation and subsequent relapse among a cohort of injection drug users in Baltimore, MD, 1988–2000. Drug Alcohol Depend..

[bib0210] Skidmore C.A., Robertson J.R., Robertson A.A., Elton R.A. (1990). After the epidemic: follow up study of HIV seroprevalence and changing patterns of drug use. BMJ.

[bib0215] StataCorp (2009). Stata Statistical Software: Release 11.

[bib0220] Stimson G., Oppenheimer E. (1982). Heroin Addiction: Treatment and Control in Britain.

[bib0225] Sweeting M., De Angelis D., Ades A., Hickman M. (2009). Estimating the prevalence of ex-injecting drug use in the population. Stat. Methods Med. Res..

[bib0230] Termorshuizen F., Krol A., Prins M., van Ameijden E.J.C. (2005). Long-term outcome of chronic drug use: The Amsterdam Cohort Study among drug users. Am. J. Epidemiol..

[bib0235] Termorshuizen F., Krol A., Prins M., Geskus R., van den Brink W., van Ameijden E.J.C. (2005). Prediction of relapse to frequent heroin use and the role of methadone prescription: an analysis of the Amsterdam Cohort Study among drug users. Drug Alcohol Depend..

[bib0240] Therneau T. (2012). coxme: Mixed Effects Cox Models. http://CRAN.R-project.org/package=coxme.

[bib0245] Turner K., Hutchinson S., Vickerman P., Hope V., Craine N., Palmateer N. (2011). The impact of needle and syringe provision and opiate substitution therapy on the incidence of hepatitis C virus in injecting drug users: pooling of UK evidence. Addiction.

[bib0250] Vaillant G. (1973). A 20-year follow-up of New York nacotic addicts. Arch. Gen. Psychiatry.

[bib0255] van den Berg C., van Brussel G., Coutinho R.M.P. (2007). Full participation in harm reduction programmes is associated with decreased risk for human immunodeficiency virus and hepatitis C virus: evidence from the Amsterdam Cohort Studies among drug users. Addiction.

[bib0260] Vlahov D., Anthony J., Munoz A., Margolick J., Nelson K., Celentano D. (1991). The ALIVE study, a longitudinal study of HIV-1 infection in intravenous drug users: description of methods and characteristics of participants. NIDA Res. Monogr..

[bib0265] Winick C. (1962). Maturing out of narcotic addiction. Bull. Narc..

[bib0270] Winick C. (1964). The life cycle of the narcotic addict and of addiction. Bull. Narc..

[bib0275] Xia Y. (2013). Characterising Patterns of Injecting Drug Use.

[bib0280] Zinberg N., Jacobson R. (1976). The natural history of chipping. Am. J. Psychiatry.

